# Beyond the binary female/male sex classification: The impact of (trans)gender on the identification of human remains

**DOI:** 10.1007/s00414-024-03348-3

**Published:** 2024-10-07

**Authors:** L. Küppers, B. Gahr, S. Ritz

**Affiliations:** Institute of Legal Medicine, University Clinic Dusseldorf, Duesseldorf, Germany

**Keywords:** Self-determination, Transgender, Gender in civil registration, Identification

## Abstract

**Abstract:**

In cases of unidentified deceased persons, sex determination is a routine task in forensic medicine. However, the binary biological sex categories ‘female’ and ‘male’ may be challenged if it is not clear whether the information in the missing persons databases refers to the biological sex or the (felt and lived) gender. An umbrella term for people who do not identify with their birth sex (which usually is the biological, chromosomal sex) is ‘transgender’. In recent decades, the legal and social situation of transgender people has changed in many countries making it easier to live their felt gender more openly. This development highlights the issue of potential challenges in the postmortem identification of transgender individuals. Serious problems in corresponding cases may be rare—but they must be considered and addressed in forensic practice to minimize the risk of delayed or failed identification. The impact of (trans)gender on the identification of human remains was examined by a narrative literature review under special consideration of the prevalences of transgender identities in general populations and in the group of unidentified deceased; possible actions to avoid problems in the postmortem identification of transgender persons in forensic practice are being proposed. One can assume that 1 of 200 people in the United States, the European Union and comparable societies is transgender with an opposite-sex identification, and 2 to 3 of 100 people live outside the typical female/male binary, with numbers increasing. If legally possible, an increasing number of transgender individuals will change their name and gender in civil registration. Transgender individuals are likely to be overrepresented in suicides and in victims of homicides. Although there are no precise data on the prevalence of transgender individuals in the group of unidentified deceased, the remarkably high reported prevalence in the general population and the over-representation of transgender individuals in suicides and homicides suggest that the topic is relevant to forensic practice. An autopsy does not always provide evidence of transgender identity, for example in skeletal remains. Particularly in unsolved cases, the possibility that an unidentified person may have been transgender should be considered. Knowledge and awareness of forensic practitioners on this topic should be strengthened by research and training. Databases and data reporting should be optimized. Recording in antemortem databases should clearly distinguish between ‘biological sex’ and ‘apparent sex /lived gender identity’. When collecting postmortem data, a clear distinction should be made between “chromosomal sex” and “sex based on morphological findings”.

**Clinical trial number:**

Not applicable (review article).

## Introduction

The question ‘female or male?’ is routinely asked when an unknown deceased person needs to be identified. Traditionally, this question relates to biological sex and follows a ‘binary conceptualisation of sexual variation’ [1].

While the physical anatomy and its underlying genetic and biochemical basis define biological sex, ‘gender’ also includes the behavioural, psychological, and social characteristics of women and men—‘gender’ is what individuals identify with and what they feel about femaleness and maleness. There is a wide range of gender identities, and it is difficult to clearly demarcate or classify them. People who do not identify with their sex assigned at birth (which—apart from rare exceptions such as intersexuality—usually is the biological, chromosomal sex) are referred to as ‘transgender’, ‘gender diverse’ or ‘gender nonconform’. A considerable proportion of these people live outside the typical female/male binary; they are ‘non-binary’, ‘gender fluid’ or ‘genderqueer’ [2–4].

In April 2024, a new law was passed in the German Bundestag which aims to strengthen self-determination of people with a gender identity that does not correspond to the attribution ‘female’ or ‘male’ assigned to them at birth. According to the ‘Law on self-determination with regard to gender entry’ (in German: ‘Selbstbestimmungsgesetz’), every citizen in Germany will be able to choose their gender and their first name and change them in a comparably simple procedure at the registration office [5, 6]. The law will come into force in November 2024 [7]. Comparable laws already exist in Argentina, Chile, Malta, Denmark, Luxembourg, Belgium, Ireland, Portugal, Iceland, New Zealand, Norway, Uruguay, Switzerland, Spain and Finland [5]. In the United States, legal recognition of transgender individuals varies by state; as of 2021, 26 states allowed for sex to be changed on birth certificates without surgery, 22 states demanded surgery and 2 states did not allow birth certificates to be altered at all [8].

This development highlights the issue of potential challenges in the postmortem identification of transgender individuals. Basically, the identification process is based on the comparison of data collected postmortem with the antemortem information on missing persons documented in missing persons databases. In Germany, the database used in such cases is called ‘VERMI/UTOT’ [9]. When a person is reported missing, the criminal police check the person’s data recorded by the registration office via searching for the name and/or the address to verify the information given by the witnesses. This also includes the witnesses’ information on the ‘apparent sex/gender’ of a person. There is a broad variety of possible cases and constellations if a transgender person is missing and/or the body of an unidentified transgender person is found. If the transgender status is obvious from autopsy results (for example the combination of breast implants and male genitalia [10]) and the transgender status is documented in the missing persons database, the identification process may be uncomplicated. Additionally, in cases of long-time missing persons or if there is evidence that the missing person might be found dead, the police try to collect DNA samples (or other evidence like the dental status) making it possible to verify the death of the person when the corresponding remains are found. If witnesses have stated a different ‘apparent gender’ than the missing person is registered as, this would be considered as an indication of a transgender identity of a missing person. So given the current legal situation, there is a comparably low risk of a mismatch between the information in the missing person report and the medically diagnosed sex. This risk particularly applies to cases where there is no DNA or dental status available, and the reported missing person’s name and address can’t be found in the registry office’s data (for example because the person was without permanent residence). When the new German ‘Selbstbestimmungsgesetz’ comes into force, things might change: The law contains a prohibition of disclosure, which means that a person's change of gender or name must not be revealed without their consent. If the birth sex of a person wouldn’t be disclosed to the investigating forces, an inconsistency between the reported gender out of the missing persons database and the forensic medical examination results would be likely. Nevertheless, the law provides the possibility of disclosing the birth sex under certain conditions. This regulation was introduced primarily to prohibit the misuse of the law by criminals to go into hiding and avoid prosecution by law enforcement authorities [6]. But the option of disclosure could be extended to cases of missing people since according to the law, it may be used for the fulfilment of the tasks of criminal prosecution or security authorities. To our knowledge, however, it is still unclear how the exchange of information between investigating authorities and registration office is supposed to take place. Should a special request from the police regarding a change in gender entry be necessary, a new awareness of the issue of transgender and identification would be required.

Generally, the functionality of the ‘binary nature of forensic sex estimation’ [8] with a focus on biological sex categories ‘female’ and ‘male’ may be challenged if it is unclear wether the biological or the felt gender is recorded in the missing persons database. This problem has already been addressed by a few authors [1, 8, 10], but to our knowledge and personal experience it has not yet received much attention from the investigating authorities, at least not in Germany.

In summary, problems in the postmortem identification of transgender persons may arise, if:

–the postmortem findings do not give any hint on an antemortem transgender status of a person; this may be the case if a strongly decomposed body is found and/or no gender affirming medical procedures have been carried out,
- and/or
–the antemortem data does neither contain molecular genetic data nor information about the transgender identity of a missing person.

These constellations may be rare—but they must be considered and addressed in forensic practice to minimize the risk of a delayed or failed identification process, and not least out of respect for the deceased person.

This article aims to make a further contribution to clarifying the relevance of the topic ‘transgender’ for forensic practice under special consideration of the prevalences of transgender identities in general populations and in the group of unidentified deceased. Furthermore, we would like to propose solutions for potential problems in the postmortem identification of transgender persons in forensic practice.

## Methods

A narrative literature review was performed to address the following questions:

1. What is the prevalence of transgender in general populations?
2. What is the risk of transgender individuals to die a violent death, and are they likely to be overrepresented in the group of unidentified deceased?

The following keywords were used for the search in the databases *Google Scholar* and *PubMed* (latest date: 07.05.2024):

- ‘transgender’ and ‘prevalence’ (both keywords in the title)
- ‘transgender’ and ‘violence’ (both keywords in the title)
- ‘transgender’ and ‘unidentified’/ ‘remains’ / ‘identification’ (both keywords in the title in each case)

The selection of inclusion criteria followed the intention of only extracting data that is relevant to the above named forensically relevant questions. It was not the aim of the work to provide a complete overview of the topic of transgender with its complex medical, social, socio-political, legal and ethical aspects.

Exclusion criteria were:

- publications written in other languages than English or German
- publications that didn’t meet the topic

Inclusion criteria were:

- Definition of the term ‘transgender’ at least similar to Meerwijk and Sevelius [11], who suggested: ‘Transgender people are those who identify as such and those whose current identity and sex assigned at birth differ’. – We chose this definition, since individuals who meet this definition are those most likely to live their felt gender and to change name and gender in civil registration.
- For the search ‘transgender and prevalence’: Literature reviews as well as general population-based surveys. – In this search we excluded publications with data from LGBTQIA + networks, maybe resulting in a greater prevalence of transgender people than in the general population.
- For the search ‘transgender and violence’: Here publications with data from LGBTQIA + networks were included.
- For the search ‘transgender’ and ‘unidentified’/ ‘remains’ / ‘identification’: All types of studies.

After sorting out the studies that met the exclusion criteria, two reviewers independently selected the publications that met the inclusion criteria under consideration of the abstracts. In a next step they read the full texts to identify the studies to be finally included. After inclusion of studies, data were extracted along the above-named questions.

## Results

**Fig. 1:**
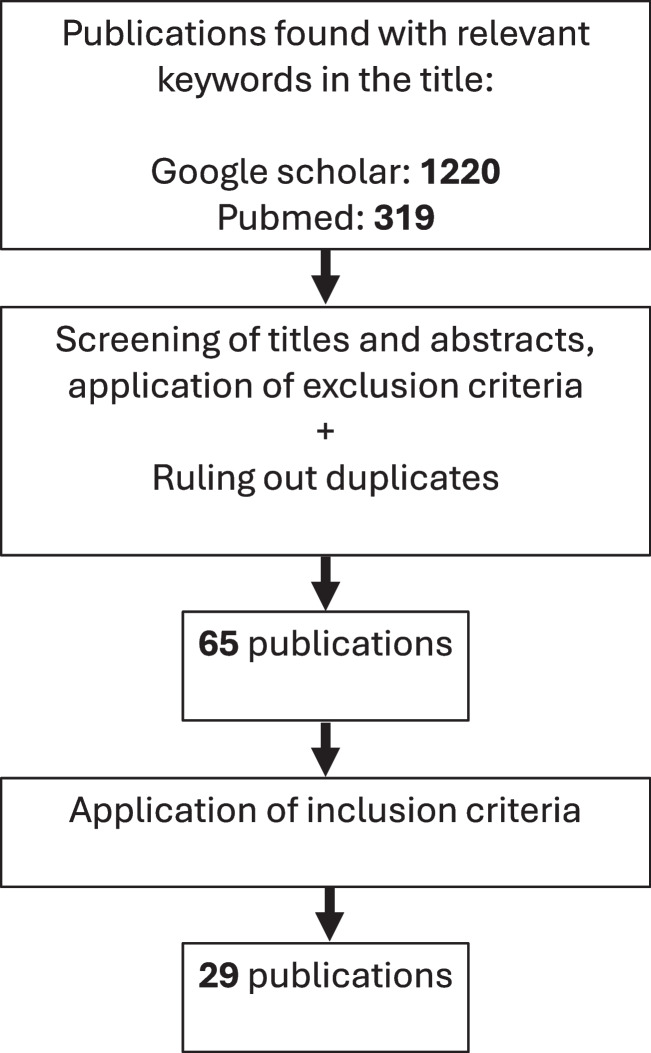
Selection of literature

Our literature search yielded 1,539 search results. After ruling out duplicates and publications that did not meet the topic or were written in another language than English or German, furthermore after application of the inclusion criteria listed above, a total of 29 publications remained (Fig. 1). These publications provided the following summarized data on the questions outlined above and were the basis for the discussion below.

### Prevalence of transgender

Participants of a 2012 Massachusetts survey (ages between 18 and 64 years, n = 28,662) identified as transgender in 0.5% of cases. The younger the participants, the more common this self-attribution was. In the 18 to 33 year old age group it was almost twice as high as in the 50 to 64 year old age group [12].

In a Dutch survey with 8064 participants, Kuyper and Wijsen (2014) found percentages of 1.1% of the participants born as a boy and 0.8% of the ones born as a girl with an incongruent gender identity (stronger identification with other sex as with sex assigned at birth) [13].

Van Caenegem et al. (2015) [14] conducted a population-based survey (n = 1832 Flemish) to determine the prevalence of gender incongruence (defined as a stronger identification with the other sex than with the sex assigned at birth) and gender ambivalence (defined as equal identification with the other sex as with the sex assigned at birth). Gender incongruence was present in 0.7% of men and 0.6% of women, gender ambivalence in 2.2% of male and 1.9% of female participants.

Based on a 2017 meta-analysis of national surveys, Meerwijk and Sevelius [11] estimated the population size of transgender people in the United States to be 0.39%. The authors point out that this estimate may be more indicative for young adults and that future surveys are likely to observe higher numbers of transgender people. Their data indicate a substantial annual increase of transgender adults in the United States.

In a 2018 study by Kaltiala-Heino and Lindberg [15], more than 3% of boys and more than 4% of girls in a population of 135.760 Finnish adolescents under the age of 21 reported their gender identity to be incongruent with the sex assigned at birth. An ‘opposite-sex identification’ was reported in 0.6% and a non-binary identification in 3.3%. The authors emphasize that these numbers (from a 2017 school-based survey) are higher than those reported in earlier comparable survey studies using data from 2008 to 2013.

According to a literature review by Nolan et al. [16], the prevalence of transgender (including transgender as well as non-binary gender) can be estimated at 0.39 – 2.7% of total US population. They reported gender conforming surgery to occur in 42–54% of trans men, 28% of trans women and 9% of persons with non-binary identities. The authors emphasize two trends, naming the increasing prevalence of transgender identities in general and a higher proportion of such identities among younger people.

### Overrepresentation of transgender persons in the group of unidentified deceased

Transgender and especially non-binary people experience more psychological distress and higher levels of anxiety and depression [17–20]. They are at higher risk for suicide [21–23] and at much greater risk of experiencing abuse and victimization [2, 11, 16, 24–27]. Transgender people also face higher levels of intimate partner violence [28–31] and may experience specific difficulties when seeking for help, for example out of fear of being stigmatized [32]. Transgender individuals are disproportionately affected by interpersonal violence and homicides with the highest risk affecting trans Black and Indigenous individuals as well as other people of color (BIPOC) [8, 33]. According to the Trans Murder Monitoring report [34], 321 trans individuals were killed worldwide between the October 2022 and September 2023. However, such data are ever only the ‘tip of the iceberg’ since they can only include deaths of confirmed trans individuals. Tallman et al. (2021) suspect that many cold cases affect trans persons [8]. Ultimately, there is no reliable data basis from which a specific prevalence of homicides against trans people could be drawn.

Since there cannot be any doubt that trans people disproportionately experience psychological distress and interpersonal violence, they are likely to be overrepresented in suicides and in victims of homicides and consecutively in the group of unidentified deceased.

## Discussion

### Relevance of the topic ‘transgender’ for the issue of postmortem identification

For a long time, large surveys aimed at general populations of states or regions did not contain questions regarding gender identity. Only recent surveys collect gender identity data more frequently and systematically [11]. Accordingly, only in the last two decades, an increasing number of studies has been published on the prevalence of transgender [11, 35]. Interpreting these data is sometimes difficult. The term ‘transgender’ is not completely uniformly defined. Since the definition of the term determines which groups are recorded as ‘transgender’ in surveys, the definition has an influence on the reported prevalences [11, 35]. Moreover, the context as well as the way how questions are asked influence the results [35]. Furthermore, the populations examined in studies often came from LGBTQIA + networks, maybe resulting in a greater prevalence of transgender people than in the general population [11].

We addressed these problems by focussing on the definition of ‘transgender’ proposed by Meerwijk and Sevelius (2017) [11] and on recent publications that present population-based data as described above.

Despite all methodological problems, the data cited appear to be very similar and suggest that one can expect prevalences of around 0.5% for opposite-sex identification and around 2–3% for a non-binary identification in the United States, the European Union and comparable societies. Another common finding is that there are higher rates of transgender individuals in younger populations. Meerwijk and Sevelius [11] suggest that the higher prevalence of transgender identities in younger populations is not due to an actual increase in the proportion of transgender identities, but it is due to people feeling more free to state that they identify as transgender.

Although it is empirically well documented that transgender people are likely to be overrepresented in suicides and in victims of homicides and consecutively in the group of unidentified deceased persons, the question of the prevalence of transgender individuals in the group of unidentified deceased cannot be answered with a specific number or percentage. Nevertheless, with the reported prevalences of around 0.5% for opposite-sex identification and around 2–3% for a non-binary identification (at least in the United States and the European Union), and under consideration of an assumed over-representation of transgender individuals in suicides and homicides, it can be concluded that the topic of transgender must have relevance for forensic practice.

Even if problems with the post-mortem identification of transgender people may only occur rarely (above all due to the availability of molecular genetic data in most cases), in these rare cases, optimal conditions for identification still need to be created. From our point of view, optimization of databases and recording as well as training and research are important goals in this context.

### Optimization of databases and reporting

One cannot assume that the investigating authorities are always aware of the topic of ‘transgender’: Waldron and Schwencke (2018) reported that in 74 of 85 deaths of transgender individuals, the abandoned name and/or gender (corresponding to the biological sex) had been used to refer to victims during investigation. The authors point out that using the abandoned name and/or gender may hinder identification and slow down an investigation [33]. When collecting data on missing people, contact persons should specifically be asked about the gender identity of the missing person. The investigation authorities should check whether entries for name and/or gender in civil databases have been changed over the course of one's life. If this is the case, information about the new name and gender should be additionally used in the identification process.

Others have already pointed out that the way transgender people are recorded in missing persons and unidentified deceased persons databases is a relevant factor [10]. Palamenghi et al. (2023) assume that a transgender person should be recorded ‘under their self-identified gender’ as this ‘reflects their life identity and is typically the gender under which their disappearance would have been reported’ [10]. This is certainly not wrong, but from the authors’ point of view, listing exclusively the self-identified gender brings with it the risk of a mismatch with unidentified remains that have been classified according to the biological sex. So, entries in antemortem databases should clearly distinguish between ‘biological sex’ and ‘apparent sex’ / ‘lived gender identity’ (according to information of the contact persons). ln the German ‘VERMI/UTOT’ database there actually are options to enter the sex of a person documented by the registration office (or determined by DNA analysis) as well as the ‘apparent sex/gender’ of a missing person [9] making mismatches during the search less likely. This seems to be a reasonable approach even if the felt gender doesn’t always have to be consistent with the outer appearance. As pointed out above, in the future it will furthermore be crucial that the information regarding a change of gender entry is revealed by the registry office to prohibit delay or failure of identification.

The importance of a clear distinction between biological sex and apparent sex/gender also applies to the recording of postmortem data. Postmortem sex diagnosis should be clearly specified (for example ‘sex by morphological findings’ or ‘chromosomal sex’). Not only would this be helpful in the identification of transgender people, but also in other, rather rare constellations, like in cases of intersexuality where chromosomal sex is inconsistent with phenotypic sex (for example a XY-women with androgen insensitivity syndrome), or in which the phenotype is not classifiable as either female or male.

### Training and research

The topic ‘biological sex and gender identity’ with its implications especially for the identification of unidentified deceased should be addressed in training and further education in the forensic field. The study of Tallman et al. (2021) impressively supports this claim by presenting survey data of forensic anthropologists; the authors critically explored the collective knowledge regarding the topic of ‘transgender’ in forensic practice and uncovered relevant gaps [8]. In general, forensic medical experts should be aware of this topic during the process of postmortem identification.

Knowledge regarding gender-affirming procedures and their detection in human remains is likely to be of particular importance. Transgender individuals have many medical options for a transition from male to female or female to male. These options include for example hormone replacement therapy (HRT), genital gender-confirmation surgery, facial feminization surgery (FFS) and facial masculinization surgery (FMS) [1, 8]. Consequences of HRT and surgical interventions can easily be determined if a corpse is well-preserved, but not in a state of skeletonization. The long-term effects of HRT on the skeleton are poorly understood [8, 10]. Based on current knowledge, reliable evidence of HRT cannot be expected when examining bones. Findings after many surgical interventions (as hairline lowering, brow lift, cheek enhancement or lip lift) will no longer be detectable in skeletal human remains. However, some FFS modifications may be detectable on the skull, especially in the glabellar region, at the mandibular or the zygomatic bones. In addition to osteosynthetic material, bone changes such as bone apposition areas can provide information [8]. Palamenghi et al. (2023) described such bone apposition areas as well as fragments of gelatinous material interpreted as residues of breast implants [10]. But even though the number of trans individuals undergoing surgical modifications grows [1], not all transgender individuals opt for surgical procedures. In a sample of 432 transgender people in Ontario [4], 30% were living their daily lives in their birth gender, 23% were living in their felt gender without medical interventions and 47% were living in their felt gender with at least some medical intervention. Only around one quarter of the MTFs (24%) and around one third of the FTMs (30%) had undergone transition-related surgical procedures. Knowledge regarding gender affirming procedures and their detection in human remains is important, so as not to overlook evidence of a transgender identity. At the same time, it is also important to be aware that the absence of such evidence does not speak against a transgender identity during lifetime.

In terms of research, working on the following scientific topics could optimize the way we deal with the challenges outlined above:

- What is the prevalence of violence against transgender individuals?—Structured surveys, inclusion of gender identity in crime statistics.
- What is the prevalence of transgender individuals within the group of unidentified deceased and of victims in cold cases?
- What are the effects of new legal regulations like the German ‘Selbstbestimmungsgesetz’ on the process of postmortem identification?
- Are there awareness and knowledge about the topic of ‘transgender’ in the context of postmortem identification? Do the investigating authorities as well as forensic medical experts consider the topic during their work? Do they need training?
- What are the effects on HRT and gender affirming surgery on the skeleton?

## Conclusion

In their 2015 publication, Scheim and Bauer question ‘whether there is any need for sex/gender designation on Canadian identification, as sex/gender is rarely used for identity verification and serves no legal purpose’ [4]. This might be true for the living, but certainly not for the dead.

Information about the birth sex as well as about the lived gender identity of a person may *both* be important for the identification process. However, until today, the process of identification has focused almost exclusively on biological sex. Even if it only affects rare cases, a rethink is necessary.

Many societies are dealing with the issue of gender diversity and transgender more and more openly, and thus provide a framework for gender diverse and transgender individuals to live their lives more freely. It can be expected that in more open societies (such as in the United States or the European Union) name changes and changes of gender entry as well as gender affirming medical procedures will increase. Even if the prevalence of transgender individuals in the unidentified deceased group cannot be determined with certainty, awareness of the possibility that a deceased had lived as a transgender person is relevant. Ultimately, it's about clarifying each individual case. So, we agree with Schall et al. (2020) [1], when they conclude: ‘…it is imperative that forensic anthropologists consider the possibility that an unidentified individual could be transgender, and not limit their analyses and conclusions to binary sex categories’ and state: ‘It is our responsibility to be aware of, and on the lookout for, nonconforming gendered individuals for whom positive identification will prove particularly problematic. Being respectful of the deceased includes being respectful of their identity in both life and death.’

## Data Availability

Data sharing is not applicable to this article as no new data were created.
